# Alterations in Red Blood Cell Functionality Induced by an Indole Scaffold Containing a Y-Iminodiketo Moiety: Potential Antiproliferative Conditions

**DOI:** 10.1155/2016/2104247

**Published:** 2016-08-29

**Authors:** Angela Scala, Silvana Ficarra, Annamaria Russo, Davide Barreca, Elena Giunta, Antonio Galtieri, Giovanni Grassi, Ester Tellone

**Affiliations:** ^1^Dipartimento di Scienze Chimiche, Biologiche, Farmaceutiche ed Ambientali, Università di Messina, Viale F. Stagno d'Alcontres 31, 98166 Messina, Italy; ^2^Virologia e Microbiologia AOOR Papardo-Piemonte, Viale F. Stagno d'Alcontres, 98166 Messina, Italy

## Abstract

We have recently proposed a new erythrocyte-based model of study to predict the antiproliferative effects of selected heterocyclic scaffolds. Starting from the metabolic similarity between erythrocytes and cancer cells, we have demonstrated how the metabolic derangement induced by an indolone-based compound (DPIT) could be related to its antiproliferative effects. In order to prove the validity of our biochemical approach, in the present study the effects on erythrocyte functionality of its chemical precursor (PID), whose synthesis we reported, were investigated. The influence of the tested compound on band 3 protein (B3), oxidative state, ATP efflux, caspase 3, metabolism, intracellular pH, and Ca^2+^ homeostasis has been evaluated. PID crosses the membrane localizing into the cytosol, increases anion exchange, induces direct caspase activation, shifts the erythrocytes towards an oxidative state, and releases less ATP than in normal conditions. Analysis of phosphatidylserine externalization shows that PID slightly induces apoptosis. Our findings indicate that, due to its unique features, erythrocyte responses to exogenous molecular stimuli can be fruitfully correlated at structurally more complex cells, such as cancer cells. Overall, our work indicates that erythrocyte is a powerful study tool to elucidate the biochemical/biological effects of selected heterocycles opening considerable perspectives in the field of drug discovery.

## 1. Introduction

Red blood cells (RBCs) are by far the most abundant cells in the blood and the simplest cells found in mammals. Due to the uniqueness of the direct relationship with each type of cell soma and owing to a metabolism greatly limited compared to other cells, RBC has become an unmatched and efficient model of scientific studies in biochemical and clinical researches [1, 2]. Its availability, the easy handling and preparation, and its natural “dispersion" in buffered aqueous solvents make it suitable to study the effects of exogenous substances on its functionality. The RBCs responses to exogenous molecular stimuli, if properly evaluated, can clarify their intriguing and seemingly simple metabolism and, at the same time, they can also be profitably correlated at structurally more complex cells, such as neoplastic cells.

**Figure 1:**
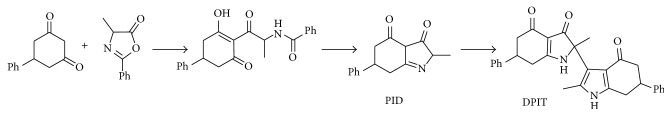
Multistep synthesis of PID.

In this scenario, we have recently proposed an unprecedented erythrocyte-based biochemical approach focused on the metabolic similarity between cancer cells and RBCs to predict the antiproliferative effects of heterocyclic scaffolds [3]. Thus, we have investigated how the metabolic derangement of RBCs induced by DPIT (2,2′-dimethyl-6,6′-diphenyl-6,6′,7,7′-tetrahydro-H,1′H-2,3′-biindole-3,4,4′(2H,5H,5′H)-trione) (Figure 1), selected as an indole-based* model compound*, could be related to its antiproliferative effects [4, 5]. With the aim to demonstrate the versatility and applicability of our approach, we herein further expand our studies focusing on PID (Figure 1), the chemical precursor of DPIT. PID is an unprecedented indole-3,4-dione synthesized by some of us* via* one-pot acid-promoted* N*-deprotection-cyclization of the corresponding 1,3,3′-tricarbonyl precursor, powerful intermediate bearing an intriguing triketo Y-topology, the latter being obtained by microwave-mediated nucleophilic addition of 5-phenyl-1,3-cyclohexanedione to 4-methyl-2-phenyl-oxazol-5-one (Figure 1) [4, 6, 7]. Actually, our interest in the chemistry of both enolizable cyclic 1,3-diketones and azlactones as building blocks for the synthesis of novel molecular architectures is well documented [4–15]. PID is a small weight heterocycle functionalized with the nitrogen analogue of the Y-triketo moiety, which could experience prototropic changes and bestows on it fascinating properties, such as an intrinsic stability and ability to act as bidentate chelating ligand.

Within our ongoing effort to propose new* N,O*-heterocycles with useful biological properties [7, 12–14], congeners of PID have been recently evaluated* in vitro* for antiviral activity against herpes simplex virus type-1 (HSV-1), resulting in lack of cytotoxicity and significant antiproliferative activity [7]. Nowadays we became interested in exploring the effect of PID on RBC functionalities, because we supposed that it could be able to cross the erythrocyte membrane, unlike its precursor, due to its smaller molecular weight, and consequently it could induce a pronounced metabolic derangement, one of whose most striking manifestations is the caspase 3 activation.

Caspase 3 belongs to a family of cysteine aspartate proteases responsible for degradation of cellular proteins and for the triggering of the apoptosis cell suicide program. It is a dormient proenzyme maintained in an inactive structural conformation, by an Asp-Asp-Asp regulatory tripeptide named “safety catch” [16]. This tripeptide is kept by* in situ* ionic interactions highly sensitive to pH that are disrupted by intracellular acidification, resulting in enhanced autocatalytic maturation of the protein that becomes more available to proteolytic activation. Resistance of caspase 3 activation plays a critical role in determining the sensitivity of cells to apoptosis and thus may contribute to the attenuated apoptosis observed in many cancers. Indeed, neoplastic cells have been shown to sequester caspase 3 in its inactive form, and thus, therapies that focus on activating caspase 3 are a promising novel anticancer strategy. The “safety catch” therefore is an important regulatory checkpoint that precludes the accidental activation of procaspase 3 in healthy cells having stable pH_i_, while facilitating proteolytic activation of caspase 3 in damaged or stressed cells in which homeostatic maintenance of normal pH_i_ is perturbed [16].

The primary cellular targets of caspase 3 are the cytoplasmic domain of the B3 (cdB3), the Na^+^/H^+^ exchanger (NHE1), and the 4 plasma membrane Ca^2+^-ATPase (PMCA4) [17–19]. As it is known, cdB3 has several functions as the maintenance of anion homeostasis, the cytoskeleton cell shape, and the regulation of the metabolic glucose 6 phosphate pathways. In detail, cdB3 competitively binds both hemoglobin (Hb) and a number of glycolytic enzymes (GE). The cleavage of cdB3 induced by caspase 3 activation causes a preferential channeling of glucose 6 phosphate (G6P) in the Embden-Meyerhof pathway (EMP) at the expense of the pentose phosphate pathway (PPP). Consequently both the increased lactate production by EMP and the lack of NADPH lead to cytosolic acidification and increase of oxidative stress [20].

NHE1 is a member of a family of electroneutral exchangers ubiquitously expressed that play an essential role in the regulation of pH_i_, protection against cytosolic acidification, and absorption of HCO_3_^−^ [21]. NHE1, activated by a decrease in pH_i_, mediates the exchange of intracellular H^+^ with extracellular Na^+^, while HCO_3_^−^ comes out from B3 in exchange for Cl^−^. NHE could be affected by numerous endogenous and exogenous stimuli and in diverse pathological situations; it has also been shown to play an important role in the proliferation disorders [22, 23].

Taking into account that intracellular alkalinization is a common feature of proliferative processes [24], Izumi et al. rightfully proposed that the induction of intracellular acidification using, for example, pharmacological inhibitors of the NHE might serve as a therapeutic tool for treating some types of cancer [25].

Pászty et al. identified an additional cleavage target of caspase 3 on the PMCA, a calmodulin-regulated Ca^2+^ pump driven by ATP expressed in the plasma membrane of all eukaryotic cells [19]. Among the multiple isoforms of PMCA, 1 and 4 are typical of RBCs [26]. PMCA4 cleavage causes irreversible activation of the Ca^2+^ transport activity of the enzyme [27]. Several studies have suggested that changes in intracellular Ca^2+^ homeostasis play an important role in apoptosis [28, 29]. Indeed, the primary switch in the decision between necrosis and apoptosis depends on various factors, including the intensity of the insult, the degree of the initial Ca^2+^ overload, and the intracellular ATP levels [30]. Deregulated apoptosis has been implicated in the development of many pathologic conditions, including neurodegenerative disorders, autoimmune diseases, sepsis, and particularly cancer [31, 32]. In this context, it is now widely acknowledged that evasion of apoptosis is one of the hallmarks of cancer development, and naturally, this discovery has led to a diverse array of scientific explorations to identify drug targets and develop compounds that might effectively treat cancer through restoration of the apoptotic program [33–36].

Altogether these considerations prompted us to investigate the influence of PID on RBC functionalities, with particular reference to caspase activation, B3, oxidative state, intracellular ATP concentration and transport, metabolism, intracellular pH, and Ca^2+^ homeostasis with the aim to point out that the metabolic derangements induced in RBC by PID would be unfavorable to the life cycle of neoplastic cells.

## 2. Materials and Methods

### 2.1. Reagents and Compounds

All reagents were purchased from Sigma-Aldrich (St. Louis, MO, USA). Citrate fresh human blood was obtained from informed healthy donors who declared that they had abstained from all drug treatment for at least one week prior to sample collection, in accordance with the principles outlined in the Declaration of Helsinki. Concentrated stock solution was prepared by dissolving PID in dimethyl sulfoxide (DMSO). PID was synthesized as previously described [4].

### 2.2. Preparation of Erythrocytes

Citrate blood samples were washed three times with an isoosmotic NaCl solution and treated as previously reported [37].

### 2.3. High Performance Liquid Chromatography (HPLC) Determinations

Washed RBCs were incubated at 37°C for 2 h with PID (100 *μ*M) in the incubation buffer (35 mM Na_2_SO_4_, 90 mM NaCl, 25 mM HEPES [N-(2-hydroxyethyl)-piperazine-N′-2-ethanesulfonic acid], and 1.5 mM MgCl_2_), adjusted to pH 7.4. Samples were washed and the packed cells were lysed with 10% ethanol. Lysates were centrifuged at 4000 ×g for 10 min at 4°C and the supernatant was filtered with 0.45 *μ*m filter. Free PID was analyzed by HPLC with a Shimadzu system, consisting of an LC-10AD pomp system and an SPDM10A diode array detector, a Rheodyne 7725i injector with a 20 *μ*L sample loop, and a reverse-phase Supelco C18 column (5 mm, 250 × 4.6 mm). The mobile phase consisted of a linear gradient of acetonitrile in H_2_O as follows: 5–20% (0–2 min), 20–30% (2–4 min), 30–100% (4–7 min), and 100% (7–10 min). The flow rate was 1.0 mL/min at 25°C. PID was detected at 286 nm and determined by comparison of peak areas with a standard solution of PID (100 *μ*M). To establish the amount of PID in the membrane bilayer, we induced RBCs hemolysis with hypotonic shock and centrifuged the samples at 15000 rpm for 15 min at 4°C. The supernatant was removed and the packed membrane structures were washed and centrifuged, as described above, three times with isoosmotic NaCl solution to eliminate the unbounded compound. After that, the packed membranes were treated with DMSO for 2 h and analyzed by HPLC to identify and quantify PID.

### 2.4. Binding to Hb

Purified Hb (0.7 mg/mL) in the T or R state was incubated for 1 h at 37°C in 0.1 M HEPES buffer plus 0.1 M NaCl with PID (100 *μ*M) and 0.3 M 2,3-biphosphoglyceric acid at pH 7.4. The free PID has been separated from the one bound to hemoglobin utilizing Microcon YM 30 (Nominal Molecular Weight Limit 30,000), filtered with 0.45 *μ*m filter, and analyzed by HPLC as described above.

### 2.5. Met-Hemoglobin (Met-Hb) Determination

Washed RBCs were treated with PID (100 *μ*M), at different incubation times from 6 to 24 h, lysed with distilled water and freezing at −20°C, and then centrifuged at 18000 rpm for 30 min. The percentage of met-Hb was determined spectrophotometrically in a range of wavelength from 500 to 680 nm.

### 2.6. Metal Chelating Activity

The chelation of Fe^2+^ by PID (0–100 *μ*M) was estimated by method of Dinis et al. [38]. The percentage inhibition of ferrozine-Fe^2+^ complex formation was calculated as [(*A*_0_ − *A*_*s*_ )/*A*_0_] × 100, where *A*_0_ was the absorbance of the control and *A*_*s*_  was the absorbance of the samples in the presence of PID (562 nm).

### 2.7. Band 3 Anion Exchanger Activity Determination: Sulphate Transport Measurement

Cells were incubated in the incubation buffer containing sulphate at 25°C, in the presence and absence of PID (100 *μ*M). At specified intervals 10 *μ*mol of 4-acetamido-4′-isothiocyanostilbene-2,2′-disulfonic acid (SITS) stopping medium was added to each test tube containing the RBC suspension. Cells were separated from the incubation medium by centrifugation (J2-HS Centrifuge, Beckman, Palo Alto, CA, USA) and washed three times at 4°C with a sulphate-free medium. After the final washing, the packed cells were lysed with perchloric acid (4%) and distilled water and centrifuged at 4°C. Sulphate ions in the supernatant were precipitated by adding glycerol and distilled water (1 : 1), 4 M NaCl and 1 M HCl solution, and 1.23 M BaCl_2_·2H_2_O to obtain a homogeneous barium sulphate precipitate. The intracellular sulphate concentration was measured by spectrophotometry at 425 nm wavelength as reported previously [39].

### 2.8. Determination of Phosphatase PTP-1B Activity

Cells were incubated in the incubation buffer at 37°C in the presence and absence of PID (100 *μ*M) and treated as previously reported [40].

### 2.9. Effects on Superoxide Anion Generation

Superoxide anions were measured as previously reported [41].

### 2.10. Reduced Glutathione (GSH) Measurements

GSH was analyzed in haemolysate using the Ellman method [42]. The samples were treated with trichloroacetic acid (TCA) and the protein precipitate was removed by centrifugation. The concentration of GSH was estimated in mmol/packed cells (PC).

### 2.11. Total Thiols Measurements

The content of the total thiols was measured using the method of Ellman [42]. Samples were diluted with a 20 mmol/L phosphate buffer, pH 8.0, containing SDS. Following this, DTNB (5,5′-dithiobis (2-nitrobenzoic acid)) from a 10 mmol/L stock solution was added and samples were incubated for 1 h at 37°C. The thiols reacted with DTNB to form anions with a strong yellow color which were optically active at 412 nm. The basal optical activity of the samples was measured before the addition of DTNB. A calibration curve was prepared using different concentrations of GSH. The concentration of the thiol groups was calculated and expressed as *μ*mol/mg proteins of plasma or as nmol/mg proteins of RBC membranes.

### 2.12. Glutathione Peroxidase (GPx) Analysis

GPx activity inside the RBC was analyzed by a commercial kit (Glutathione Peroxidase Cellular Activity Assay Kit, Sigma-Aldrich) following the instruction supplied by the seller.

### 2.13. Lipid Peroxidation Assay

Isolated RBCs were incubated for 2 h in the absence or in the presence of PID (25, 50, and 100 *μ*M) and analyzed as previously described [3].

### 2.14. Acetylcholinesterase (AChE) Enzyme Assay

AChE activity was assayed in RBCs suspensions after PID (100 *μ*M) treatment using the colorimetric method proposed by Ellman et al. [43].

### 2.15. Measurement of Percentage Haemolysis

The haemolysis of RBCs was determined spectrophotometrically at 576 nm based on the ratio of Hb released from cells to the total cellular Hb content after haemolysis with distilled water. The ratio of haemolysis was calculated from the following equation: *H*(%) = *A*_1_/*A*_2_ × 100%, where *H*(%) is the percent of haemolysis of the RBCs, *A*_1_ is the absorbance of the supernatants of the samples of the RBCs incubated with or without PID (100 *μ*M), and *A*_2_ is the absorbance of the supernatant of the samples after complete haemolysis with distilled water.

### 2.16. Caspase 3 Assay

Citrate blood samples were washed three times with an isoosmotic NaCl solution and treated as previously reported [41], using PID (50 *μ*M) and tert-butyl-hydroperoxide (t-BHT), 100 *μ*M.

### 2.17. pH_i_ Measurement

Isolated RBCs were incubated from 2 to 24 h in the absence or in the presence of PID (100 *μ*M). After incubation, the samples were washed 3 times with 10 volumes of isoosmotic NaCl and lysed by treatment with ice distilled water, vortex, and ultrasonication. Then the samples were centrifuged and the pH_i_ was measured using a pH meter ProLab 3000 Schott.

### 2.18. Annexin V Apoptosis Detection

Fluorescence-activated cell sorting (FACS) analysis was performed as described by Andree et al. [44]. RBCs were incubated for 6, 12, and 24 h in the presence or absence of PID (100 *μ*M) in annexin-binding buffer containing 0.14 M NaCl, 0.01 M HEPES-NaOH (pH 7.4), and 2.5 mM CaCl_2_. RBCs were suspended in a solution composed of Annexin-V-Fluos and annexin buffer. After 10 min of incubation in the dark, samples were finally diluted 1 : 5 in annexin-binding buffer and measured using flow cytometric analysis. Cells were analyzed by forward scatter, and annexin fluorescence intensity was measured in fluorescence channel FL-1 with an excitation wavelength of 488 nm and an emission wavelength of 530 nm.

### 2.19. Measurement of Intra-/Extracellular Ca^2+^

Isolated RBCs were incubated for 2 and 6 h in the absence or in the presence of PID (100 *μ*M). After incubation, the samples were washed 3 times with 10 volumes of isoosmotic NaCl and centrifuged at 2500 rpm for 5 min. Then, intra-/extracellular Ca^2+^ concentration was analyzed by a commercial kit (Calcium Colorimeric Assay Kit, Sigma-Aldrich) following the instruction supplied by the seller.

### 2.20. Measurement of Intra-/Extracellular ATP

ATP was measured by the luciferin-luciferase technique, as previously reported [45].

### 2.21. Statistical Analysis

Data are presented as mean of four different experiments ± standard deviation (SD). The data were analyzed by one-way analysis of variance. The significance of the differences in relation to the respective controls for each experimental test condition was calculated by Student's *t*-test for each paired experiment. A *P* value of <0.05 versus control was regarded as significant difference and indicated with asterisks in the figures.

## 3. Results and Discussion

### 3.1. PID Crosses the RBC Membrane

**Figure 2:**
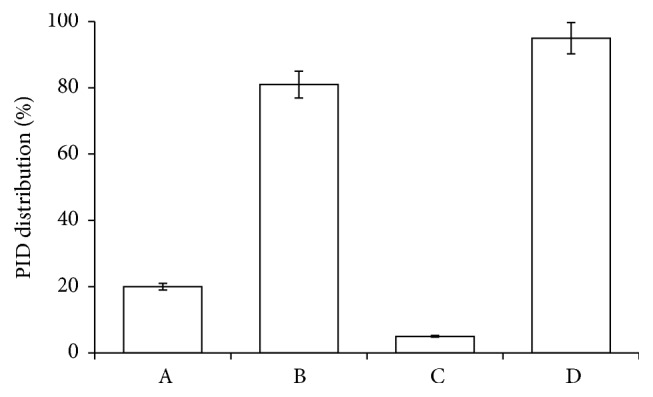
HPLC determination: PID distribution inside (A), outside (B), and in the RBC membrane (C), compared to the control (PID 100 *μ*M standard solution (D)).

HPLC observations of RBCs incubated with PID (100 *μ*M) at 37°C for 2 h reveal that PID crosses the RBC membrane reaching inside of the cell a 20% share (Figure 2). The different molecular weight could probably explain the greater ability of PID to cross the plasma membrane, localizing in the cytosol, with respect to the precursor DPIT. According to our previous reports [36, 40, 41, 46, 47], we can extrapolate that exogenous compounds permeate more easily through the RBC membrane within the molecular weight range 200–300 g/mol.

### 3.2. PID Does Not Bind Hb but Increases the B3 Protein Exchange

Since Hb and B3 are the two most abundant RBC proteins, inside the cytoplasm and in the membrane, respectively, the effects of PID on their structure and functionality were explored.

To this end, purified Hb was incubated with PID (100 *μ*M) for 1 h at 37°C and the levels of free PID were assessed by HPLC, excluding PID-Hb interaction (data not shown). Furthermore it does not affect the Hb redox reactions because no increased values of met-Hb were registered incubating RBCs with PID (100 *μ*M) for 6-12-24 h (data not shown). Additionally, the inability of PID to chelate Fe^2+^ was demonstrated by UV-vis spectroscopy.

**Figure 3:**
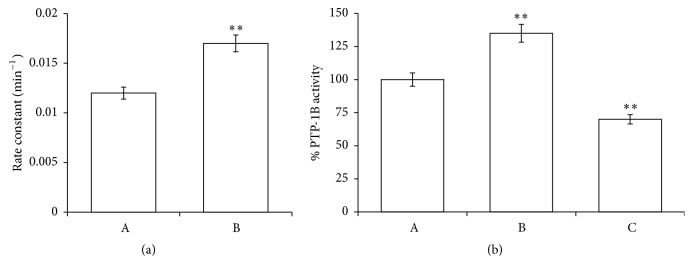
Effects of PID on rates of sulphate transport (a) and on phosphatase activity (b) in normal human RBCs, incubated in absence (A) or in the presence of 100 PID *μ*M (B) or OV 1.0 mM (C). Results are from four independent experiments ± standard deviation. Asterisks indicate significant differences at *P* < 0.05 versus control.

The influence of PID on B3 was studied evaluating spectrophotometrically its effect on anion exchanger functionality after pretreatment of RBCs with PID (100 *μ*M) and comparing the results with the control.  Figure 3(a) shows an increase of anion exchange of about 30% in the presence of PID (rate constant: 0.017 and 0.012 min^−1^ in RBCs incubated with and without PID, resp.).

The derangement of B3 function, being one of the main causes of the pH_i_ decrease, could act as a factor which creates an “acidic environment” for organ cells. Since pH_i_ has been shown to be alkaline in many human cancer cells and to be an important trigger for cell proliferation [24], PID influence on B3 functionality could contribute to inhibiting cell proliferation and leading the tumor cells to be more sensitive to antitumor drug. Also the fact that B3 interacts with and regulates the function of p16, a key negative regulating protein for the cell cycle [48], is not to be underestimated. In this context Shen et al. demonstrated that B3 plays a crucial role in the pathogenesis of gastric and colonic adenocarcinoma and that p16 dysfunction is a novel pathway of carcinogenesis [49]. To find potential justification for PID-induced destabilization on B3 physiological exchange, we tested the tyrosine phosphatase activity as an index of phosphorylation state of RBC (Figure 3(b)). It is noteworthy that changes in phosphorylation are among the most important modulations of protein activity in RBCs [50]. In particular, the delicate balance between phosphorylation and dephosphorylation on RBC membrane depends on the action of two types of proteins, tyrosine phosphatases (PTP1B) and src tyrosine kinases that are strongly influenced by free radical concentration [51].

Thus, the PTP1B activity was tested in the presence of PID (100 *μ*M) or orthovanadate (OV), a known phosphatase inhibitor. Results shown in Figure 3(b) highlighted that PID induced hyperactivation of phosphatases (about 30%) in comparison to the control, clearly indicating an alteration of RBC phosphorylation balance.

The phosphorylation and consequent inhibition of the pyruvate dehydrogenase complex (PDC) would contribute to the Warburg metabolic correlated with malignant progression of cancer cells [52]. Taking into account the metabolic similarities between RBCs and cancer cells that we have recently proposed [3], we can speculate that the correction of this metabolic abnormality could offer opportunities for cancer treatment and may potentially synergize with other cancer therapies.

### 3.3. PID Influences the RBC Oxidative State

**Figure 4:**
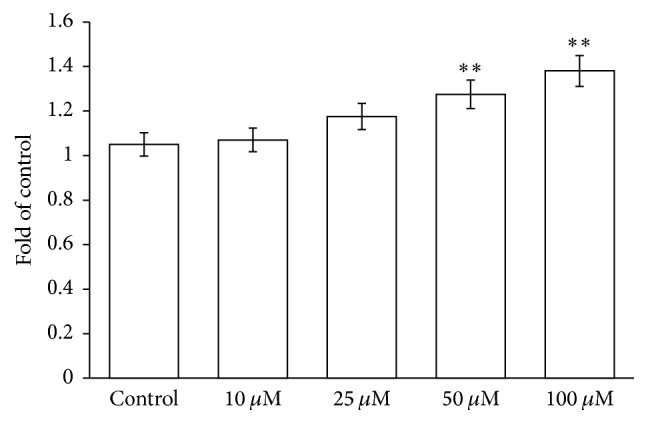
Effects of PID (0–100 *μ*M) on superoxide anion radical generation. Results are from four independent experiments ± standard deviation. Asterisks indicate significant differences at *P* < 0.05 versus control.

The influence of PID on the redox equilibrium of the RBCs was evaluated in terms of superoxide generation, GSH levels, GPx activity, thiol redox status, and lipid peroxidation. The rate of superoxide generation was analyzed* in vitro* at different concentrations (10, 25, 50, and 100 *μ*M), resulting in the fact that PID triggered superoxide generation at 50 *μ*M and more evidently at 100 *μ*M (Figure 4).

**Figure 5:**
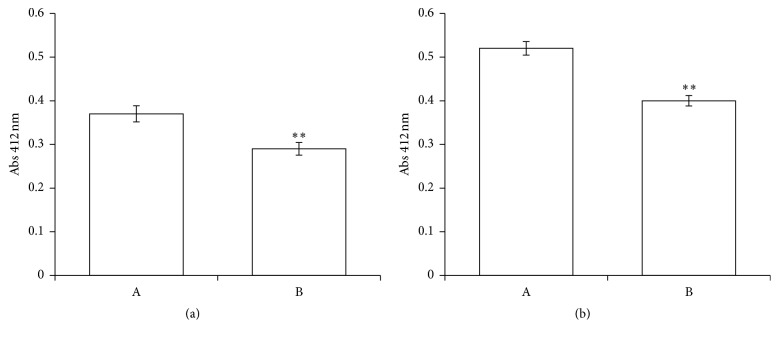
Influence of PID on intracellular levels of GSH (a) and total thiols (b). The RBCs were incubated for 2 h in absence (A) or in the presence of PID 100 *μ*M (B). Results are from four independent experiments ± standard deviation. Asterisks indicate significant differences at *P* < 0.05 versus control.

GSH is a principal intracellular thiol-containing compound and it is involved in maintaining the oxidation-reducing balance in RBCs. Therefore GSH concentration and thiol redox status have been evaluated in RBCs pretreated with PID (100 *μ*M).  Figure 5 shows the depletion of GSH (a) and the decrease of -SH groups (b), in comparison to the control. Furthermore, the GPx activity was also tested, showing that PID does not alter the enzyme functionality (data not shown).

**Figure 6:**
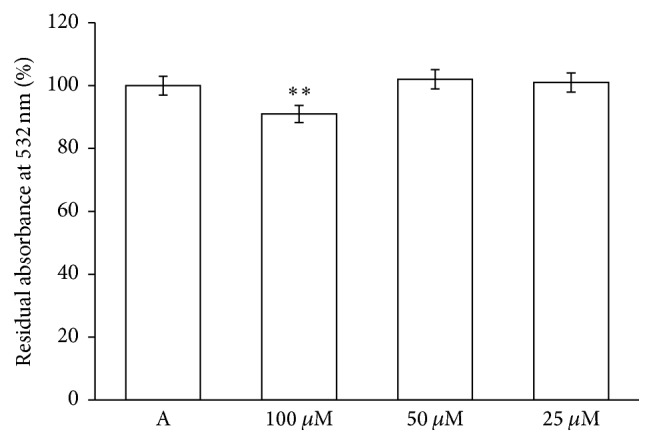
Influence of PID on lipid peroxidation of RBC membrane. The RBCs were incubated for 2 h in absence (A) or in the presence of PID (25–100 *μ*M). Results are from four independent experiments ± standard deviation. Asterisks indicate significant differences at *P* < 0.05 versus control.

Oxidation of -SH groups is strictly related with lipid oxidation of the membrane. Then peroxidation was evaluated on the RBC membrane after incubation for 2 h with PID (25-50-100 *μ*M). Unexpectedly a slight inhibition at the higher concentration was observed, compared with the control (Figure 6), likely due to the ability of PID to break the lipid peroxidation chain reaction.

Such considerations are further supported by the evaluation of the integrity of plasma membrane assessed monitoring the functionality of AChE, a well-known marker of cell membrane wholeness, resulting in the fact that PID (0–100 *μ*M) did not significantly modify the enzyme activity (data not shown). Moreover, it did not increase the percentage of haemolysis.

Based on the experimental evidences, we assume that PID shifts the RBCs towards an oxidative state, increasing the generation of superoxide and the oxidation of thiol groups. Additionally, reduced GSH levels are detected, leading to dangerous oxidant/antioxidant imbalance and to an increase of intracellular H_2_O_2_. Indeed GSH, through the action of GPx, catalytically detoxifies the cells from peroxides such as H_2_O_2_. So the GSH depletion always causes accumulation of reactive oxygen species (ROS) and consequently intracellular acidification [24, 53].

### 3.4. PID Influences Caspase 3 Activation and pH_i_

**Figure 7:**
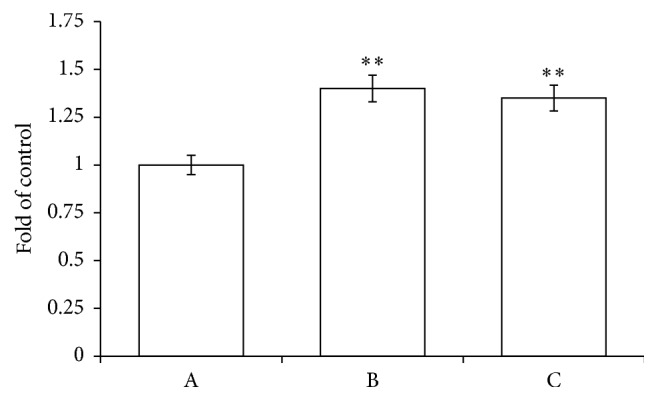
Caspase 3 activity in RBCs in the absence (A) and in the presence of PID 50 *μ*M (B) or t-BHT 100 *μ*M (C). Results are from four independent experiments ± standard deviation. Asterisks indicate significant differences at *P* < 0.05 versus control.

Generally, the increase of the oxidative stress and the decrease of pH significantly contribute to the direct activation of caspase 3 by removal of the “safety catch” [16, 54–56]. To confirm the above, RBCs were incubated, respectively, in the absence and in the presence of PID (50 *μ*M) or t-BHT (100 *μ*M) as a reference oxidant.  Figure 7 shows that PID significantly induces caspase 3 activation, even superior to t-BHT.

Generally, caspase 3 activation leads to inappropriate triggering or rapid disablement of key structural proteins and important signaling, homeostatic and repair enzymes [57]. In nucleate cells, caspase 3 processing occurs in a protease cascade involving mitochondrial release of cytochrome c in the cytosol, while in RBCs, in the absence of mitochondria and cytochrome c, this mechanism appears to operate directly. Caspase 3 catalyzes the specific cleavage of cdB3, NHE1, and PMCA4. The cdB3 and NHE1 cleavage contributes to the alteration of the hydrogen ions concentration, as HCO_3_^−^/Cl^−^ exchange occurs in conjugation with the Na^+^/H^+^ antiporter [18]. The hyperstimulation of the B3 induced by PID should be offset by the NHE1 activity to maintain the correct pH homeostasis. However, caspase 3 activation results in NHE1 inhibition and reduced Na^+^/H^+^ antiporter activity acidifies cells [18, 58]. Therefore, PID would change the pH_i_ of RBCs inducing cytosolic acidification, according to literature [59]. To confirm the above, pH_i_ was measured by incubating RBCs in the presence of PID (100 *μ*M), resulting in a decrease of 0.1 units. Literature data have recently reported that intracellular acidification in mammalian cells, typically amounting to 0.3–0.4 pH_i_ units, can be detected following exposure of cells to external stimuli as UV irradiation, staurosporine, and etoposide [24]. Our experimental observation, namely, a variation of 0.1 pH_i_ units could be related to the presence in RBCs of a high concentration of Hb that can buffer a more pronounced cytosolic acidification. Indeed, Hb, at a concentration of 7 mmoles per litre of cell water, is the RBC's main proton buffer [60].

Furthermore, within the last decade, numerous studies have demonstrated that pH_i_ homeostasis is often dramatically altered in cancer cells, as they maintain a pH_i_ more alkaline than their normal counterparts [61]. This has sparked substantial interest in pH regulation as a potential therapeutic target relevant to many forms of cancers [62]. In particular, regulation of pH_i_ may be a possible mechanism for tumor-selective therapy. Rightfully, it has even been proposed that the induction of an intracellular acidification, using, for example, pharmacological inhibitors of the NHE, might serve as a therapeutic tool for treating some types of cancer. In this context, we postulate that PID may determine in cancer cells a more pronounced cytosolic acidification with respect to that observed in our “buffered” erythrocyte-based model of study, providing a way of inducing tumor-specific apoptosis, thus aiding cancer chemotherapy.

### 3.5. PID Influences Intracellular Ca^2+^ Homeostasis, ATP Efflux, and RBC Metabolism

**Figure 8:**
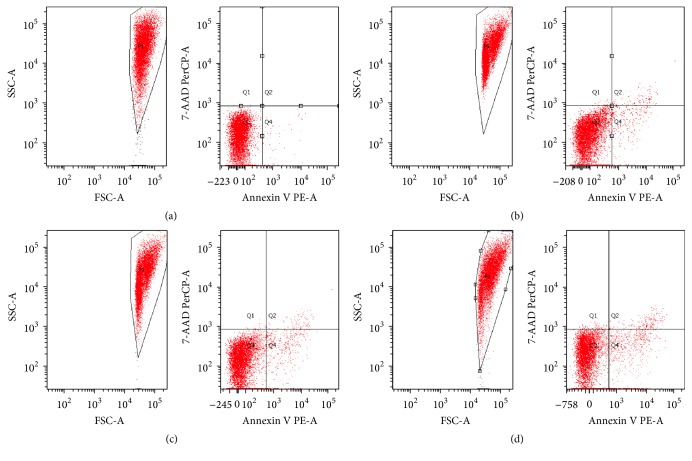
Flow cytometric analysis of apoptosis in RBC in the absence (a) and in the presence of PID (100 *μ*M) after 6 h (b), 12 h (c), and 24 h (d) of incubation time.

Generally, a decrease in pH_i_ is the initial trigger for a cascade of events resulting in apoptosis [25]. Indeed, acidification facilitates the caspase 3 activation by removal of the “safety catch”, that in turn has been shown to induce phosphatidylserine (PS) exposure [63]. In normal RBCs, plasma membranes exhibit significant phospholipid asymmetry, with phosphatidylcholine and sphingomyelin predominantly on the external side and phosphatidylethanolamine and PS on the inner side. Entry into apoptosis leads to a loss of phospholipid asymmetry, with exposure of PS on the outer side. It was shown that the anticoagulant annexin V preferentially binds to negatively charged phospholipids like PS. Thus, this binding of annexin V was used to detect PS exposure on the membrane of apoptotic cells in cytofluorimetric assays (Figure 8). Our experiments were performed at 6, 12, and 24 h, resulting in a 4.2% of apoptosis in the early phase (6 h), while at longer incubation periods the intensity of the apoptotic process increases (4.8% at 12 h; 8.2% at 24 h).

Triggers of apoptosis include exposure to several stressors such as oxidative stress, NHE inhibitors, cytosolic acidification, and increase of cytosolic Ca^2+^ levels [64]. In this regard, the effects of PID (100 *μ*M) on the calcium-permeable channels PMCA were evaluated at 2 h and 6 h, showing a slight increase in the intracellular free Ca^2+^ levels (data not shown), in accordance with the low apoptotic effect observed.

**Figure 9:**
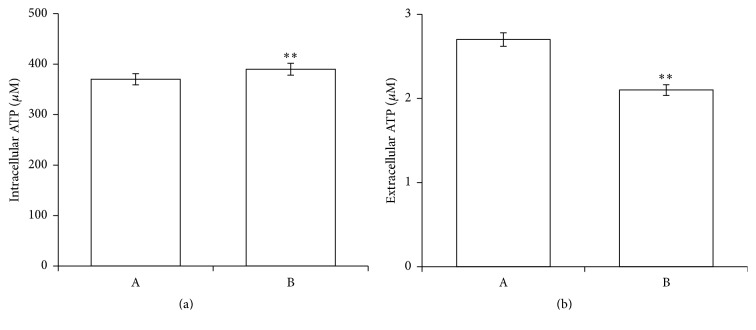
Effect of PID on the intracellular (a) and extracellular (b) ATP levels in RBCs. ATP concentrations were measured at the end of the incubation time of erythrocytes without (A) and with PID 100 *μ*M (B). Results are from four independent experiments ± standard deviation. Asterisks indicate significant differences at *P* < 0.05 versus control.

The limiting factor of the PMCA transport capacity is ATP availability [50]. Indeed, both the Ca^2+^ homeostasis and the cellular ATP are important determinants of cell death. In particular, cells remain alive when certain level of ATP is maintained, but when ATP falls below this level, apoptosis is activated, and a severe drop in cellular ATP causes cell necrosis [65]. Thus, the influence of PID on ATP release from RBCs was evaluated, showing that RBCs pretreated with PID (100 *μ*M) released significantly less ATP than in normal conditions, but the intracellular [ATP] does not appear affected by the treatment (Figure 9), although both the PMCA4 hyperactivity and the phosphatases triggering should deplete the cellular ATP.

We suggest that this condition could be related to an alteration of the metabolic modulation of RBCs attributable to PID influence. In particular, as cdB3 serves as a docking station for multiple GE, its cleavage operated by caspase 3 deprives RBCs of the fundamental and primary regulation of metabolic G6P pathways. Specifically, the predominant EMP is favored to produce ATP and NADH, at the expense of the PPP, only source of reducing power (NADPH). Cancer cells experience a substantial need of reducing power in the form of NADPH for the biosynthesis of lipids and nucleotides required during proliferation. In this context, PID would interfere with cell proliferation not only by reducing NADPH and GSH availability, but also by positively modulating the functionality of pyruvate kinase M2 (PKM2) isoform expressed in cancer cells with low activity [66].

In particular, both the EMP enhancement, with the wider availability of fructose 1,6 biphosphate, and the cytosolic acidification induced by PID would activate the PKM2. Thus, the use of small molecule PKM2 activators may be an appropriate approach to interfere with cancer cell metabolism for therapeutic purposes.

**Figure 10:**
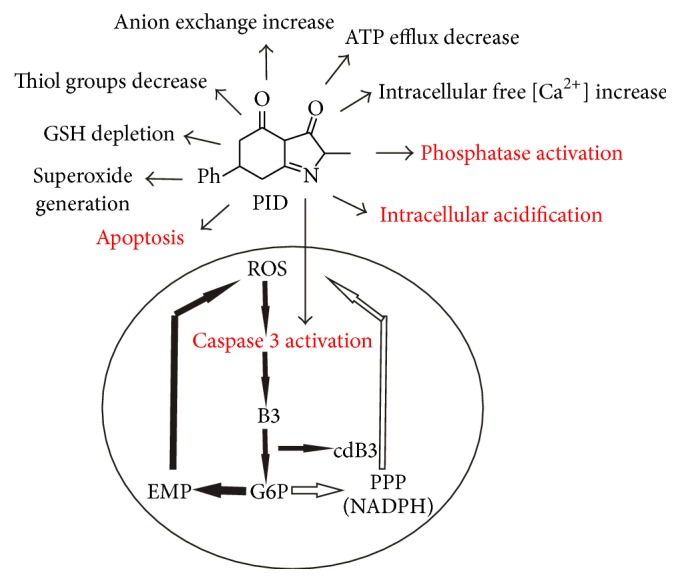
Effects of PID in RBCs and schematic representation of the “vicious circle” induced by PID. In red, the main antiproliferative conditions are highlighted.

In summary, this study contributes to highlighting the great potentiality of RBCs as versatile cellular model of study to predict the antiproliferative behaviour of selected heterocycles with different cellular localization. In particular, DPIT [3] is almost completely intercalated in the phospholipid bilayer, while PID crosses the RBC membrane. This different distribution leads to a series of complex metabolic responses that can be due to direct interactions/activations with cytosolic components and consequent increment of endogenous oxidative stress (i.e., PID) or to extracellular signals trigger that, on the whole, can culminate in the same increase of oxidative stress (i.e., DPIT). In particular, the effects of PID on RBCs, culminating in the caspase activation, would be represented in a “vicious circle” (Figure 10), in which the main antiproliferative conditions are highlighted.
